# Comparison of Two Commercially Available qPCR Kits for the Detection of *Candida auris*

**DOI:** 10.3390/jof7020154

**Published:** 2021-02-22

**Authors:** Janko Sattler, Janina Noster, Anne Brunke, Georg Plum, Pia Wiegel, Oliver Kurzai, Jacques F. Meis, Axel Hamprecht

**Affiliations:** 1 Institute for Medical Microbiology, Immunology and Hygiene, University Hospital of Cologne, University of Cologne, 50935 Cologne, Germany; janko.sattler@uk-koeln.de (J.S.); anne.brunke@rub.de (A.B.); georg.plum@uk-koeln.de (G.P.); pia.wiegel@uk-koeln.de (P.W.); 2 German Centre for Infection Research, Partner Site Bonn-Cologne, 50937 Cologne, Germany; 3 Institute for Medical Microbiology and Virology, Carl von Ossietzky University Oldenburg, 26129 Oldenburg, Germany; janina.noster@uol.de; 4 Institute for Hygiene and Microbiology, University of Würzburg, 97080 Würzburg, Germany; okurzai@hygiene.uni-wuerzburg.de; 5 German National Reference Centre for Invasive Fungal Infections, Leibniz Institute for Natural Product Research and Infection Biology—Hans Knöll Institute, 07745 Jena, Germany; 6 Center of Expertise in Mycology Radboud University Medical Center/Canisius Wilhelmina Hospital, 6532 SZ Nijmegen, The Netherlands; jacques.meis@gmail.com; 7 Department of Medical Microbiology and Infectious Diseases, Canisius Wilhelmina Hospital, 6532 SZ Nijmegen, The Netherlands

**Keywords:** qPCR, detection limits, sensitivity, strain specificity, commercial kits, *Candida auris*, Fungiplex *Candida Auris*, *Auris*ID

## Abstract

*Candida auris* is an emerging pathogen with resistance to many commonly used antifungal agents. Infections with *C. auris* require rapid and reliable detection methods to initiate successful medical treatment and contain hospital outbreaks. Conventional identification methods are prone to errors and can lead to misidentifications. PCR-based assays, in turn, can provide reliable results with low turnaround times. However, only limited data are available on the performance of commercially available assays for *C. auris* detection. In the present study, the two commercially available PCR assays *Auris*ID (OLM, Newcastle Upon Tyne, UK) and Fungiplex *Candida Auris* RUO Real-Time PCR (Bruker, Bremen, Germany) were challenged with 29 *C. auris* isolates from all five clades and eight other *Candida* species as controls. *Auris*ID reliably detected *C. auris* with a limit of detection (LoD) of 1 genome copies/reaction. However, false positive results were obtained with high DNA amounts of the closely related species *C. haemulonii, C. duobushaemulonii* and *C. pseudohaemulonii*. The Fungiplex *Candida Auris* RUO Real-Time PCR kit detected *C. auris* with an LoD of 9 copies/reaction. No false positive results were obtained with this assay. In addition, *C. auris* could also be detected in human blood samples spiked with pure fungal cultures by both kits. In summary, both kits could detect *C. auris*-DNA at low DNA concentrations but differed slightly in their limits of detection and specificity.

## 1. Introduction

*Candida* spp. are the most common causative agents of fungal infections in humans, ranging from local skin colonisation to blood stream infections with high mortality rates [1]. Whereas the most common species *C. albicans* can usually be successfully treated with antifungal agents, other species such as *C. auris*, *C. glabrata*, and *C. krusei* may be less susceptible or resistant to antifungals including different azoles or amphotericin B [2,3,4,5,6].

*C. auris* was first reported in 2009 and infections are particularly challenging to treat [7]. Besides the limited therapeutic options, *C. auris* shows high transmission rates in nosocomial environments [8] regularly leading to hospital outbreaks, possibly due to effective biofilm formation on biotic and abiotic surfaces [9]. These two characteristics—multidrug resistance and effective transmission—underline the need for rapid and reliable detection of *C. auris* in clinical samples.

Misidentification of pathogens in routine laboratories can have serious consequences for medical treatment and outcome, especially in case of strain-specific virulence properties [10]. In particular, conventional microbiology techniques are prone to errors. *C. auris* shows strong sequence homology to other *Candida* species like *C. lusitaniae*, *C. haemulonii*, *C. pseudohaemulonii* etc. [11,12,13], which leads to high rates of misidentifications [14,15,16]. Biochemical identification of *C. auris* isolates by the commercial system API ID 32C (Biomérieux) resulted in misidentification as *C. sake* or *C. intermedia* in 83% or 17% of the samples tested, respectively [17]. Even with matrix-assisted laser desorption/ionisation time-of-flight (MALDI-TOF) correct identification of *C. auris* was not achieved in all cases [17], often due to incomplete databases [16,18]. Furthermore, both biochemical and MALDI-TOF based identification require previous cultivation of the pathogen, which may lead to delayed detection [19].

Molecular methods, in turn, could rapidly detect *C. auris* directly from clinical samples. By sequence analysis and specific primer design, adequate detection and differentiation of *C. auris* from other *Candida* species using PCR techniques has been achieved [20]. Nevertheless, the establishment and validation of protocols using in house primers are time consuming and require deep methodological knowledge. Therefore, several commercially available and user-friendly qPCR kits for the detection of *C. auris* have been developed. So far, data on the performance of these kits is scarce. In this work the performance of the kits Fungiplex *Candida Auris* RUO Real-Time PCR kit (Bruker, Bremen, Germany) and *Auris*ID (OLM, Newcastle Upon Tyne, UK) was evaluated, using genomic DNA of 29 molecularly characterized *C. auris* isolates from all five different clades as well as DNA from eight other *Candida* species.

## 2. Material and Methods

The commercial qPCR kits *Auris*ID (OLM, Newcastle Upon Tyne, UK) and Fungiplex *Candida auris* RUO Real Time PCR (Bruker, Bremen, Germany; further referred to as Fungiplex CaRT) were challenged with genomic DNA from pure cultures of 29 molecularly characterized *C. auris* isolates [17,21,22] (Table S1). The challenge collection comprised isolates of all five clades with the following distribution: clade I: 17, clade II: 2, clade III: 5, clade IV: 4, clade V: 1. One isolate of each of the closely related *Candida* species *C. pseudohaemulonii, C. haemulonii and C. duobushaemulonii* as well as two isolates each of more distantly related *Candida* species (*C. albicans*, *C. glabrata*, *C. tropicalis*, *C. krusei* and *C. parapsilosis*) were additionally tested to determine the specificity of the tests.

Isolates were cultured on CHROMagar *Candida* (Mast Group, Reinfeld, Germany) at 37 °C for 24 h. Subsequently, DNA was isolated using the DNeasy UltraClean Microbial Kit (Qiagen, Hilden, Germany) according to the manufacturer’s instructions. A baseline dilution of 10 ng DNA/µL was created for each sample, as determined by NanoDrop One (Thermo Fisher Scientific, Waltham, MA, USA). Genome copies were calculated using the formula copy number = (amount DNA [ng] * 6.022 × 10^23^)/(length [bp] * 1 × 10^9^ * 650) and a genome size of 12.1 Mb for *C. auris* [13].

To determine the specificity of the assays, DNA of the control samples was tested with both kits at concentrations of 60 ng DNA/reaction (ca. 5 × 10^6^ copies/reaction). In case of false positive results, serial 10-fold dilutions were tested up to the dilution level at which correct negative results were obtained in two out of two independent runs using DNA originating from the same extraction.

To determine the limit of detection (LoD), serial 10-fold dilutions of DNA, starting with ca. 5 × 10^6^ genome copies/reaction were examined for all isolates. If two out of two or two out of three independent runs showed positive results, the respective isolate was counted as positive for the genome copy number of this dilution. Replicates were run with DNA originating from the same extraction. The limit of detection was calculated as previously described by Forootan et al. [23]. Briefly, based on the number of positive isolates at the respective copy number, a replicate standard curve was generated using Prism 8.1 (GraphPad, San Diego, CA, USA) and the LoD threshold determined which gave 95% positive PCR results (Figure S1).

To assess the impact of human DNA on the tests, human blood was spiked with a suspension of *C. auris.* Starting from a suspension equivalent to a 0.5 McFarland standard, a 10-fold dilution series of this suspension was prepared up to a dilution of 1:10^4^ and added to human blood at a ratio of 1:10. Preliminary experiments showed that this equals roughly concentrations from 10 to 100,000 colony forming units (CFU)/mL. Exact CFU/mL concentration was determined by plating 100 µL of the spiked blood samples on Columbia Blood Agar and Malt Extract Agar plates and counting the colonies after 48 h incubation at 37 °C. DNA was extracted from 200 µL of the respective spiked blood samples plus two pure blood samples using the QIAamp DNA Mini Kit (Qiagen). DNA was eluted in 200 µL buffer and used for qPCR runs without further dilution steps. All qPCR runs were performed on an ABI7500 Real-Time PCR system (Applied Biosystems, Foster City, CA, USA). Amplification protocols are shown in Table S2.

## 3. Results

### 3.1. Evaluation of Species Specificity

To assess specificity, both kits were challenged with high amounts of genomic DNA (ca. 5 × 10^6^ copies/reaction) extracted from five *C. auris* isolates of all five clades. Furthermore, three isolates of the closely related species *C. haemulonii*, *C. duobushaemulonii* and *C. pseudohaemulonii* as well as 10 isolates of the more distantly related species *C. albicans*, *C. glabrata*, *C. krusei*, *C. parapsilosis* and *C. tropicalis* were tested as controls.

All five *C. auris* samples were correctly identified as positive by both assays. While no false-positive results were obtained with the Fungiplex CaRT kit and *Auris*ID for less related *Candida* species, samples of *C. haemulonii*, *C. duobushaemulonii* and *C. pseudo-haemulonii* gave rise to false-positive results with *Auris*ID. Serial dilutions of genomic DNA were used to determine thresholds for false positivity. When a maximum of ca. 5 × 10^5^ copies/reaction for *C. haemulonii* and *C. pseudohaemulonii* and ca. 5 × 10^4^ copies/reaction for *C. duobushaemulonii* were employed, no false-positive results were recorded (Table S3).

### 3.2. Determination of Detection Limits

**Figure 1:**
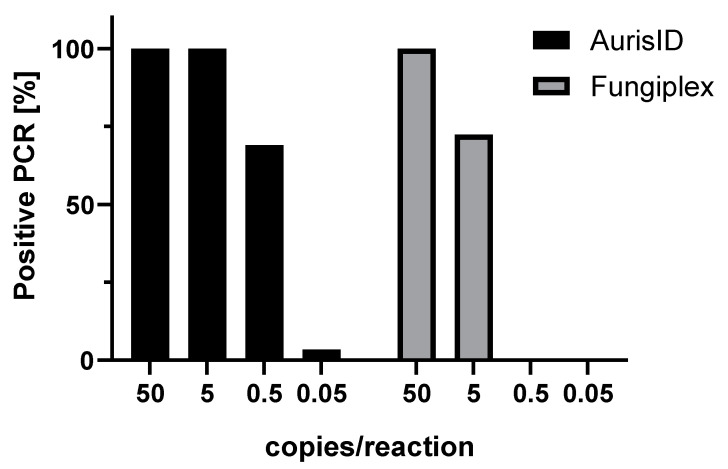
Detection rates of *C. auris* by *Auris*ID and Fungiplex CaRT. The assays were challenged with DNA of 29 *C. auris* isolates at various copy numbers.

To determine the detection limits of the assay, 29 *C. auris* isolates were tested in at least two replicates using both kits (Figure 1). Whereas *AurisI*D identified all strains as *C. auris*-positive at DNA amounts of ca. 5 copies/reaction or lower, Fungiplex CaRT detected all tested samples at amounts of ca. 50 copies/reaction and 72% of all isolates (21/29) at 5 copies/reaction. At further dilutions equivalent to ca. 0.5 or 0.05 copies/reaction, *Auris*ID was positive in 69% (20/29) or 3% (1/29) of all samples, while no positive results were obtained with Fungiplex CaRT. Based on these results the LoD was calculated as 1 copy/reaction for *Auris*ID and 9 copies/reaction for Fungiplex CaRT (Figure S1).

To determine the impact of human DNA on *C. auris* detection, DNA extracted from *C. auris* cultures of two isolates (381 and 382) and DNA from human blood were combined at a ratio of 1:10. Serial dilutions of the DNA mixture were tested with the PCR assays. The Fungiplex CaRT kit detected both isolates at amounts of ca. 50 copies/reaction and the *Auris*ID at ca. 5 copies/reaction (Table 1), indicating no significant inhibition effect of human DNA on the assay.

For further evaluation of the performance on clinical samples, human blood was spiked with a dilution series of the same two *C. auris* isolates and DNA was extracted directly from the spiked samples. *C. auris* could be detected by both kits in a dilution dependent manner. Human DNA and blood components did not interfere with *C. auris* detection. Comparable to assays from pure *C. auris* cultures, detection limits for the two spiked blood samples were around 10-fold higher for Fungiplex CaRT compared to *Auris*ID (ca. 32/45 CFU/reaction for Fungiplex CaRT and 3/2 CFU/reaction for *Auris*ID) (Table 1). Furthermore, pure blood samples did not lead to false-positive results or inhibition of the PCR assays.

### 3.3. Impact of C. auris Clade on Performance

Since *C. auris* clades could impact detection by PCR, obtained data was additionally analysed after stratification by clades (Table S4). 

Interestingly, at ca. 5 copies/reaction 82% (14/17) of the samples of clade I were tested positive with Fungiplex CaRT compared to 25% (1/4) of the samples in clade IV. However, this difference did not reach statistical significance (*p* = 0.0526 by Fisher’s exact test). No correlation between clade and detection limit was observed at any other concentration for both assays (Table S4).

## 4. Discussion

To the best of our knowledge, this is the first systematic evaluation of two commercially available PCR based kits for *C. auris* detection, the *Auris*ID and Fungiplex CaRT. *Auris*ID demonstrated higher sensitivity with a LoD of 1 copy/reaction compared to 9 copies/reaction for Fungiplex CaRT. At very high amounts of ca. 5 × 10^6^ copies/reaction or 5 × 10^5^ copies/reaction, *Auris*ID gave rise to false-positive results for closely related *Candida* species, in contrast to Fungiplex CaRT. Likely, this is of little diagnostic relevance as clinical specimen contain much lower amounts of *C. auris* DNA compared to purified DNA from colonies.

When analysing spiked blood samples, a similar difference in the detection limit of the two kits was observed. The results obtained with these commercially available kits show good sensitivity as described in previous studies with in house assays using Taq-Man chemistry-based PCR [24]. The authors determined a detection limit of 1 CFU/reaction. High sensitivity was also proven for the commercial kit GPS^TM^ MONODOSE CanAur dtec-qPCR kit (Alicante, Spain), which yielded positive results for samples with 5 to 10 copies of the DNA template [25]. In the present study, LoDs of 1 copy/reaction (*Auris*ID) and 9 copies/reaction (Fungiplex CaRT) were recorded and 2/3 CFU/reaction (*Auris*ID) and 32/45 CFU/reaction (Fungiplex CaRT) in the spiked blood experiments. However, a comparison of the data to the aforementioned studies is difficult given the varying efficiency of DNA extraction and a different experimental setup.

The commercially available kits assessed in the present study may reduce hands-on-time even for inexperienced users due to easy-to-follow protocols. In addition, commercially available assays allow high reproducibility and consistency [25].

*Auris*ID provided false-positive results for closely related *Candida* species when high DNA concentrations of ca. 5 × 10^6^ and 5 × 10^5^ copies/reaction were used. It should be noted that the recommended cycling protocol had to be changed because the ramp rates of the ABI platform used were not sufficiently high. Therefore, the annealing/synthesis step was extended from 20 to 30 s, which could have increased unspecific primer binding. Therefore, the assay was additionally run on the Rotor-Gene Q cycler (Qiagen, Hilden, Germany), which could be operated with the recommended protocol. However, false-positive results were obtained for the same samples and with the same DNA concentrations as with the ABI platform. Therefore, false-positive results are likely caused by less specific oligonucleotide binding of the *Auris*ID assay. While exact primer and probe sequences have not been disclosed by the manufacturers, both kits have different target regions, with the 28S ribosomal gene region for *Auris*ID and the mating locus alpha for Fungiplex CaRT. Since 28S is a multicopy target, it might explain the lower LoD of 1 copy/reaction. In contrast, the copy number of the exact mating locus alpha target used for Fungiplex CaRT is not known.

In previous studies it was shown that identification success by biochemical methods may depend on the specific clade analysed [26]. In contrast, we did not observe any significant clade-specific difference in performance with these two molecular assays. A numerically higher detection of clade I compared to clade IV by Fungiplex CaRT was noted at ca. 5 copies/reaction which should be analysed with a larger number of samples in future studies. However, for some clades, particularly clade II and V, only small numbers of samples were included in this study so that no definite conclusions can be drawn.

Some aspects that could potentially affect the final results have not been considered in our evaluation. For comparison reasons, both assays were challenged with the same amount of DNA and a higher sensitivity was recorded for *Auris*ID. However, it has to be noted that higher template volumes (10 µL instead of 6 µL) can be used for the Fungiplex CaRT assay, which will likely improve detection when working with clinical samples. In the present study, DNA extracted mainly from pure cultures was used. However, various specimen in diagnostic routine may contain microbiota, host cells or other substances, which could possibly impact the results. Since the prevalence of *C. auris* infection or colonization is very low in Germany, DNA from cultured isolates instead of clinical specimen had to be used for this evaluation. Successful detection of *C. auris* from spiked blood samples and from mixed DNA extracted from *C. auris* and human blood indicated that both assays work well in the presence of human DNA and likely for other clinical samples. However, future studies are needed to validate the performance of both assays directly on various clinical specimen and their usefulness in the hospital setting. In addition, DNA extraction methods differ in terms of efficacy as well as stability of the extracted nucleic acids and therefore also may influence the overall performance. Furthermore, DNA extraction kits used in diagnostic laboratories are usually optimized for isolation of bacterial or viral DNA. Fungal cells have a thicker cell wall and may therefore require different lysis strategies [27]. Before using any *C. auris* PCR kits on clinical samples, laboratories should assess the performance of their DNA extraction kit for clinical mycological samples.

In summary, based on our results, *Auris*ID and Fungiplex CaRT are suitable for identification of *C. auris* even at low DNA concentrations. *Auris*ID showed a higher sensitivity for *C. auris* detection and Fungiplex CaRT a higher specificity. Both assays have easy-to-follow protocols, thus facilitating reliable diagnostics of *C. auris* infections.

## Figures and Tables

**Table 1:** Performance of PCR assays on human blood. Threshold for PCR positivity for two isolates using (a) spiked blood samples and (b) mixed DNA (*C. auris* cultures/blood).

Isolate	(a) Viable CFU/Reaction for *C. auris*-Spiked Blood Samples		(b) Copies/Reaction Determined for Mixed DNA *C. auris*/Blood	
	*Auris*ID	Fungiplex CaRT	*Auris*ID	Fungiplex CaRT
381	~3	~32	~5	~50
382	~2	~45	~5	~50

## Data Availability

The data presented in this study are available in Figure 1, Tables S3 and S4. Raw data are available on reasonable request from the corresponding author.

## Supplementary Materials

The following are available online at https://www.mdpi.com/2309-608X/7/2/154/s1, Figure S1: Determination of the limit of detection for (A) AurisID and (B) Fungiplex CaRT. The LoD was calculated as the threshold which resulted in 95% positive samples, Table S1: *C. auris* isolates used in this study. BSI, blood stream infection, Table S2: qPCR protocols applied for the different kits, Table S3: Thresholds for false-positive results. Both kits were challenged with serial dilutions of DNA extracted from eight negative controls, Table S4: PCR positivity in relation to *C. auris* clade. Shown is the number of correctly identified isolates from each clade at the respective copy number.

## References

[1] Vincent J.L., Rello J., Marshall J., Silva E., Anzueto A., Martin C.D., Moreno R., Lipman J., Gomersall C., Sakr Y. (2009). International study of the prevalence and outcomes of infection in intensive care units. J. Am. Med. Assoc..

[2] Pfaller M., Diekema D. (2004). Twelve years of fluconazole in clinical practice: Global trends in species distribution and fluconazole susceptibility of bloodstream isolates of Candida. Clin. Microbiol. Infect..

[3] Vermitsky J.P., Edlind T.D. (2004). Azole resistance in *Candida glabrata*: Coordinate upregulation of multidrug transporters and evidence for a Pdr1-like transcription factor. Antimicrob. Agents Chemother..

[4] Young L.Y., Hull C.M., Heitman J. (2003). Disruption of ergosterol biosynthesis confers resistance to amphotericin B in *Candida lusitaniae*. Antimicrob. Agents Chemother..

[5] Orozco A.S., Higginbotham L.M., Hitchcock C.A., Parkinson T., Falconer D., Ibrahim A.S., Ghannoum M.A., Filler S.G. (1998). Mechanism of fluconazole resistance in *Candida krusei*. Antimicrob. Agents Chemother..

[6] Chowdhary A., Prakash A., Sharma C., Kordalewska M., Kumar A., Sarma S., Tarai B., Singh A., Upadhyaya G., Upadhyay S. (2018). A multicentre study of antifungal susceptibility patterns among 350 *Candida auris* isolates (2009–2017) in India: Role of the ERG11 and FKS1 genes in azole and echinocandin resistance. J. Antimicrob. Chemother..

[7] Kean R., Brown J., Gulmez D., Ware A., Ramage G. (2020). *Candida auris*: A decade of understanding of an enigmatic pathogenic yeast. J. Fungi.

[8] Schelenz S., Hagen F., Rhodes J.L., Abdolrasouli A., Chowdhary A., Hall A., Ryan L., Shackleton J., Trimlett R., Meis J.F. (2016). First hospital outbreak of the globally emerging *Candida auris* in a European hospital. Antimicrob. Resist Infect. Control.

[9] Chatzimoschou A., Giampani J.F., Meis E.R. (2021). Activities of nine antifungal agents against *Candida auris* biofilms. Mycoses.

[10] Zong Z., Wang X., Deng Y., Zhou T. (2012). Misidentification of Burkholderia pseudomallei as Burkholderia cepacia by the VITEK 2 system. J. Med. Microbiol..

[11] Sharma C., Kumar N., Pandey R., Meis J.F., Chowdhary A. (2016). Whole genome sequencing of emerging multidrug resistant *Candida auris* isolates in India demonstrates low genetic variation. New Microbes New Infect..

[12] Kathuria S., Singh P.K., Sharma C., Prakash A., Masih A., Kumar A., Meis J.F., Chowdhary A. (2015). Multidrug-resistant *Candida auris* misidentified as *Candida haemulonii*: Characterization by matrix-assisted laser desorption ionization-time of flight mass spectrometry and DNA sequencing and its antifungal susceptibility profile variability by Vitek 2, CLSI broth microdilution, and Etest method CL. J. Clin. Microbiol..

[13] Muñoz J.F., Gade L., Chow N.A., Loparev V.N., Juieng P., Berkow E.L., Farrer R.A., Litvintseva A.P., Cuomo C.A. (2018). Genomic insights into multidrug-resistance, mating and virulence in *Candida auris* and related emerging species. Nat. Commun..

[14] Snayd M., Dias F., Ryan R.W., Clout D., Banach D.B. (2018). Misidentification of *Candida auris* by RapID yeast plus, a commercial, biochemical enzyme-based manual rapid identification system. J. Clin. Microbiol..

[15] Kordalewska M., Perlin D.S. (2019). Identification of drug resistant *Candida auris*. Front. Microbiol..

[16] Mizusawa M., Miller H., Green R., Lee R., Durante M., Perkins R., Hewitt C., Simner P.J., Carroll K.C., Hayden R.T. (2017). Can multidrug-resistant *Candida auris* be reliably identified in clinical microbiology laboratories?. J. Clin. Microbiol..

[17] Hamprecht A., Barber A.E., Mellinghoff S.C., Thelen P., Walther G., Yu Y., Neurgaonkar P., Dandekar T., Cornely O.A., Martin R. (2019). *Candida auris* in Germany and previous exposure to foreign healthcare. Emerg. Infect. Dis..

[18] Buil J.B., van der Lee H.A.L., Curfs-Breuker I., Verweij P.E., Meis J.F. (2019). External quality assessment evaluating the ability of Dutch clinical microbiological laboratories to identify *Candida auris*. J. Fungi.

[19] Mancini N., Carletti S., Ghidoli N., Cichero P., Burioni R., Clementi M. (2010). The era of molecular and other non-culture-based methods in diagnosis of sepsis. Clin. Microbiol. Rev..

[20] Kordalewska M., Zhao Y., Lockhart S.R., Chowdhary A., Berrio I., Perlin D.S. (2017). Rapid and accurate molecular identification of the emerging multidrug-resistant pathogen *Candida auris*. J. Clin. Microbiol..

[21] De Groot T., Puts Y., Berrio I., Chowdhary A., Meis J.F. (2020). Development of *Candida auris* short tandem repeat typing and its application to a global collection of isolates. MBio.

[22] Alfouzan W., Ahmad S., Dhar R., Asadzadeh M., Almerdasi N., Abdo N.M., Joseph L., de Groot T., Alali W.Q., Khan Z. (2020). Molecular epidemiology of *Candida auris* outbreak in a major secondary-care hospital in Kuwait. J. Fungi.

[23] Forootan A., Sjöback R., Björkman J., Sjögreen B., Linz L., Kubista M. (2017). Methods to determine limit of detection and limit of quantification in quantitative real-time PCR (qPCR). Biomol. Detect. Quantif..

[24] Leach L., Zhu Y., Chaturvedi S. (2018). Development and validation of a real-Time PCR assay for rapid detection of *Candida auris* from surveillance samples. J. Clin. Microbiol..

[25] Martínez-Murcia A., Navarro A., Bru G., Chowdhary A., Hagen F., Meis J.F. (2018). Internal validation of GPS^TM^ MONODOSE CanAur dtec-qPCR kit following the UNE/EN ISO/IEC 17025:2005 for detection of the emerging yeast *Candida auris*. Mycoses.

[26] Ambaraghassi G., Dufresne P.J., Dufresne S.F., Vallières É., Muñoz J.F., Cuomo C.A., Berkow E.L., Lockhart S.R., Luong M.-L. (2019). Identification of *Candida auris* by use of the updated Vitek 2 yeast identification system, version 8.01: A multilaboratory evaluation study. J. Clin. Microbiol..

[27] Fredricks D.N., Smith C., Meier A. (2005). Comparison of six DNA extraction methods for recovery of fungal DNA as assessed by quantitative PCR. J. Clin. Microbiol..

