# Supplementary material for: Comparison of Two Commercially Available qPCR Kits for the Detection of Candida auris
Source: J Fungi (Basel). 2021 Feb 22;7(2):154. doi: 10.3390/jof7020154 (PMC7926799; doi:10.3390/jof7020154)
Supplement: Supplementary file 1 [file jof-07-00154-s001.pdf]

Comparison of two commercially available qPCR kits for the detection of *Candida auris*

Janko Sattler, Janina Noster, Anne Brunke, Georg Plum, Pia Wiegel, Oliver Kurzai, Jacques F. Meis and Axel Hamprecht

Supplementary Data

Table S 1. *C. auris* isolates used in this study. BSI, blood stream infection.

Clade	Isolate	Origin	State
I	382	BSI	India
	387	BSI	Pakistan
	388	BSI	Pakistan
	389	BSI	India
	390	BSI	India
	10081242	Skin swab	UK
	10111019	Urine	Netherlands
	10051259	BSI	India
	10081640	BSI	South Asia
	10081113	BSI	South Asia
	10081274	BSI	Kuwait
	10081273	BSI	Kuwait
	10110808	BSI	South Asia
	10110990	BSI	Montenegro
	10110211	BSI	Netherlands
	10081227	BSI	Belgium
	C10777	Skin swab	Germany
II	381	BSI	Japan
	10031062	BSI	South Korea
III	383	BSI	S. Africa
	384	BSI	S. Africa
	10051549	BSI	S. Africa
	10081199	BSI	Spain
	10081197	BSI	Spain
IV	385	BSI	Venezuela
	386	BSI	Venezuela
	10111014	BSI	Colombia
	10111015	BSI	Colombia
V	10111018	BSI	Iran

Table S 2. qPCR protocols applied for the different kits.

Kit	Activation	Denaturation	Annealing	Cycles
Fungiplex CaRT	95 °C, 15 min	95 °C, 5 s	60 °C, 30 s	45
*Auris*ID	95 °C, 2 min	95 °C, 10 s	60 °C, 30 s	40

Table S 3. Thresholds for false-positive results. Both kits were challenged with serial dilutions of DNA extracted from eight negative controls.

	**Threshold false-positive PCR (copies/reaction)**	
**Isolate/strain**	***Auris*ID**	**Fungiplex**
C. duobushaemulonii	$5x10^5$	-
C. haemulonii	$5x10^6$	-
C. pseudohaemulonii	$5x10^6$	-
C. albicans	-	-
C. glabrata	-	-
C. krusei	-	-
C. parapsilosis	-	-
C. tropicalis	-	-

Table S 4. PCR positivity in relation to *C. auris* clade. Shown is the number of correctly identified isolates from each clade at the respective copy number.

			**Isolates detected (%)**			
Clade	No. of isolates	Assay	50 copies/ reaction	5 copies/ reaction	0.5 copies/ reaction	0.05 copies/ reaction
**I**	17	*Auris*ID	17 (100%)	17 (100%)	13 (76%)	1 (6%)
		Fungiplex	17 (100%)	14 (82%)	0 (0%)	0 (0%)
**II**	2	*Auris*ID	2 (100%)	2 (100%)	1 (50%)	0 (0%)
		Fungiplex	2 (100%)	1 (50%)	0 (0%)	0 (0%)
**III**	5	*Auris*ID	5 (100%)	5 (100%)	4 (80%)	0 (0%)
		Fungiplex	5 (100%)	4 (80%)	0 (0%)	0 (0%)
**IV**	4	*Auris*ID	4 (100%)	4 (100%)	2 (50%)	0 (0%)
		Fungiplex	4 (100%)	1 (25%)	0 (0%)	0 (0%)
**V**	1	*Auris*ID	1 (100%)	1 (100%)	0 (0%)	0 (0%)
		Fungiplex	1 (100%)	1 (100%)	0 (0%)	0 (0%)

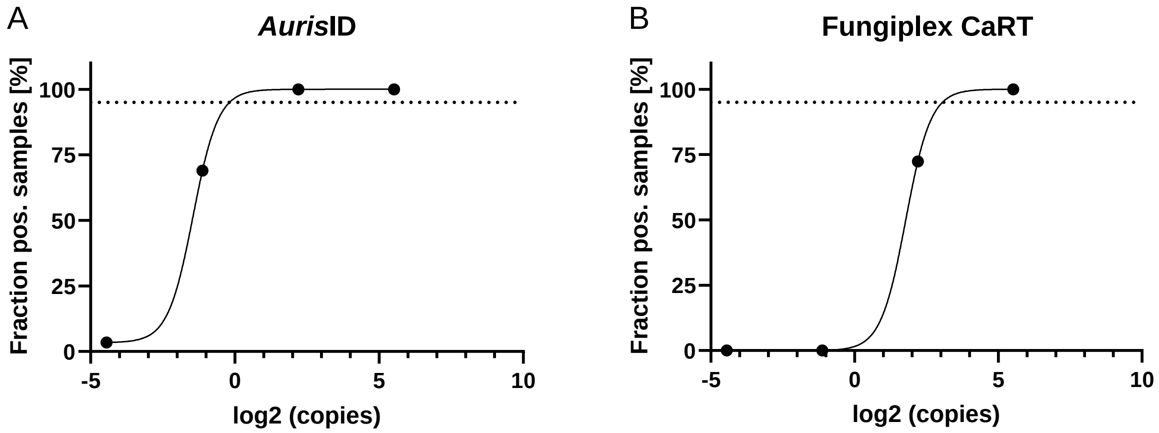

Figure S 1. Determination of the limit of detection for (A) *Auris*ID and (B) Fungiplex CaRT. The LoD was calculated as the threshold which resulted in 95% positive samples.
